# A retrospective evaluation of parental smoking and the risk of Type 1 diabetes in children

**DOI:** 10.18332/tid/195228

**Published:** 2024-11-21

**Authors:** Ipek Cicekli, Raika Durusoy

**Affiliations:** 1 Department of Nutrition and Dietetics, Faculty of Health Sciences, Acibadem University, Istanbul, Türkiye; 2 Department of Nutrition and Dietetics, Institute of Health Sciences, Acibadem University, Istanbul, Türkiye; 3 Department of Public Health, Faculty of Medicine, Ege University, Izmir, Türkiye

**Keywords:** secondhand smoke, diabetes mellitus, prevention, passive smoking, tobacco induced disease

## Abstract

**INTRODUCTION:**

The association between secondhand smoking (SHS) and the risk of Type 1 diabetes mellitus (DM) has garnered increasing interest. The aim of this study is to examine whether exposure to SHS is associated with an increased likelihood of Type 1 DM.

**METHODS:**

This study was designed as a case-control study. Children aged 4–14 years diagnosed with Type 1 DM who were followed in the Endocrine and Metabolic Diseases Outpatient Clinic were included as cases, and healthy children (without any chronic disease) in the same age range were included as the controls. A total of 248 children were included in the study, with two research arms. The structured questionnaire was applied face-to-face. Adjusted odds ratios (AOR) and 95% confidence intervals (CIs) of other risk factors were evaluated by multivariable regression analysis.

**RESULTS:**

No difference was found in the number of cigarettes mothers smoked daily and the duration of the smoking period during pregnancy and lactation, between the two groups. Among the cases, the daily number of cigarettes smoked by parents at home was 3.28 ± 4.90, higher than in the controls (p=0.039). Comparing the controls, children with Type 1 DM were more likely to be exposed to SHS at home by 1.08 (95% CI: 1.004–1.15, p=0.039) times in cases.

**CONCLUSIONS:**

Children with Type 1 DM had higher odds of being exposed to SHS at home. These results suggest substantial health gains could be made by extending effective public health interventions to reduce exposure to SHS and prevent Type 1 DM in children.

## INTRODUCTION

There is a growing interest in the association between secondhand smoking (SHS) and the risk of Type 1 diabetes mellitus (DM). Exposure to SHS occurs when non-smokers breathe smoke exhaled by people who smoke or from burning tobacco products^1^.

In addition to primary factors that promote the onset of diabetes, passive smoking is also associated with an increased risk of insulin resistance and Type 2 DM^2,3^. Exposure to SHS, especially at home, may be a risk factor for diabetes management.

Several studies show that SHS increases the risk of Type 2 DM^4^, including gestational DM^5^. It has been suggested that passive smoking not only increases the prevalence of impaired glucose tolerance (IGT) in a time-dependent manner but is also a risk factor for impaired fasting glucose, IGT, and Type 2 DM. However, the effects of passive smoking on the development of Type 1 DM are unclear^6^. Moreover, studies on Type 1 DM are insufficient and generally focus on maternal smoking during pregnancy^3,7-9^ and Type 1 DM development in offspring, or on active smoking and risk of diabetes^10^.

SHS contains the same harmful substances that smokers inhale, and there is no safe threshold for SHS exposure. Infants and young children are the most vulnerable to the harmful consequences of SHS, and are least capable of evading SHS^11^. The most of their exposure to SHS is attributed to adults (parents or others) smoking within the confines of their home. In the case of very young children, SHS also amplifies the likelihood of more severe complications, including sudden infant death syndrome^12,13^. A retrospective study of data from 192 countries underlined that as many as 40% of children are regularly exposed to SHS^13^.

Previously, we presented other risk factors associated with Type 1 DM (colostrum feeding, exclusive breastfeeding duration, total breastfeeding duration, cereals introduction, and baby formula)^14^. The present study tests the hypothesis of a relationship between SHS and Type 1 DM.

## METHODS

### Population

This study was designed as a case-control study. The cases were patients diagnosed with Type 1 DM who were aged 4–14 years and followed in the Endocrine and Metabolic Diseases polyclinic of the Ege University Pediatrics Hospital, between January and March 2020. The controls were children who came to the General Pediatric Outpatient Clinic of the same hospital, with the same age range and were not diagnosed with any chronic disease including Type 1 DM. General Pediatrics outpatient clinic admissions were due to newly developing acute conditions [the majority of the admissions were upper respiratory tract infection (65%), vitamin D deficiency (5%), and healthy child follow-up (3%)], and 85–90% were first visits to the hospital; 10–15% were invited for a follow-up one month later, so the follow-up children were also of the same age. If they have a chronic condition, they go to pediatric specialty clinics and start follow-up in those clinics.

The sample size was calculated as a minimum of 210 children, each group comprising 105 children, using the t-test group in the G*Power program for two groups, with an effect size of 0.5 (a medium anticipated difference between two groups for a scale-type variable), a margin of error of 0.05, and a power of 95%. A total of 252 volunteers were planned to be included in the study by adding 20% to this sample size to account for possible non-response, and the recruitment stopped with a total of 248 volunteers. The case group included 122 children, and the control group had 126, meeting the minimum required sample size. In the data collection process (before reaching the sample size), a total of 11 parents who did not want to participate in the study in the general pediatric outpatient clinic were excluded. In the control group, children who presented to the outpatient clinic with an acute condition but had an underlying chronic disease were also excluded from the study. Also, seven parents did not want to participate in the study because they did not want to share their information, parents of two children thought they would not remember the study questions, and two parents did not want to participate in the outpatient clinic. The response rates for all eligible cases and controls admitted during the data-collecting period were 96% and 91%, respectively.

### Questionnaire

The study questionnaire, structured by the researchers in light of the literature, was applied face-to-face. The study was explained to the children included in the study and their parents, and the questions were asked. The questions relating to the characteristics of both cases and controls (sex, age, body mass index, form of delivery, birth order, and birth interval), for cases only (duration of Type 1 DM and age at diagnosis, HbA1c levels at diagnosis were examined retrospectively from the patient file), and both groups’ maternal history of GDM and their family characteristics (family history of Type 1 DM, education level, residence), were included.

Smoking status was ascertained during pregnancy and lactation, and in the household. To evaluate a child’s smoking exposure before diagnosis of Type 1 DM, parents were asked (yes/no) about the status of smoking near the child before diagnosis, and those who answered ‘yes’ were asked for the duration of smoking and the number of cigarettes smoked in the same closed environment with the child at home. Maternal smoking status during pregnancy and lactation was asked with a response of yes/no.

As for anthropometric measures, in calculating the percentile values, reference values for Turkish children were used^15^. Body mass index (kg/m^2^) was used to assess childhood obesity. In the study, children’s BMI percentiles were classified according to the percentiles of the CDC growth chart^16^. Children with a BMI <5th percentile were considered underweight, 5th–84th percentile normal weight, 85th–94th percentile overweight, and ≥95th percentile obese.

### Measurement of SHS

Mothers were individually asked whether they smoked during pregnancy, and if so, they were further queried about their daily cigarette consumption during that time. The same set of questions was reiterated for the lactational period. Importantly, the case group (children with Type 1 DM) and the control group (healthy children) were subjected to identical inquiries during the maternal and lactational periods. Subsequently, comparisons were performed using the data collected from these specific time frames.

For the household smoking variable, parents of controls were asked about their smoking behavior when they were included in the study. However, for the cases, the inquiry occurred before the diagnosis of Type 1 DM to explore the association with SHS. After collecting data, we compared smoking-related variables between the two groups: for controls during their participation in the study and for cases before the Type 1 DM diagnosis period. Notably, we specifically questioned parents about the number of daily cigarettes smoked at home to examine children’s passive smoking status. The study refers to smoking within the home as household smoking. The exposure resulting from this household smoking is defined as secondhand smoke (SHS). Researchers also examined children’s SHS exposure during three different periods: during pregnancy, during lactation, and the at-home period.

The child’s exposure to cigarettes before the diagnosis of Type 1 DM was calculated by asking the number of cigarettes smoked per day by the parents who smoked before the diagnosis of their children.

### Statistical analyses

The data were analyzed using the PASW Statistics Version 18.0 software platform^17^. The descriptive variables of cases and controls were compared with two-tailed tests: Student’s t-test (continuous variables), Mann Whitney U test (when a non-parametric test was necessary), and chi-squared test (categorical variables). The smoking status of the parents in the case group before the diagnosis and the number of cigarettes they smoked daily, were compared with McNemar’s test and t-test for dependent groups. To be able to measure the relationship between smoking and the odds of having Type 1 DM disease, the parent’s smoking status before the diagnosis date was questioned. In the case group, the parent’s smoking status and cigarettes smoked per day before and after diagnosis (current, during data collection) were also compared.

Multivariable analysis with the enter method evaluated adjusted odds ratios (AOR) and 95% confidence intervals (CIs) of other confounders. Logistic regression analyses were conducted with the adjusted variables based on biological plausibility (age and sex). Multivariable analyses were performed with sensitivity analyses (Supplementary file Table 1) with models with probable confounders both biological variables (sex, age) and variables that were significant in univariate analysis (birth interval, family history, residence) and other probable confounders (education status) for maternal and lactational smoking status and smoking at home. In all analyses, p<0.05 was accepted as a statistical significance level.

### Ethics

Written permission was obtained from Ege University Children’s Hospital, Department of Pediatrics, in order to conduct the research in the relevant institution. Approval was obtained from the Ethics Committee for Clinical Research, Ege University, Faculty of Medicine (decision number 10059, dated 9 January 2020).

## RESULTS

The mean age of Type 1 DM diagnosis of the children in the cases included in the study was significantly less than the mean age of the control group (6.30 ± 4.03 years and 7.48 ± 2.56 years, respectively). The mean of Type 1 DM duration was 4.16 ± 3.85 years in the cases. No statistical significance was found in sex, form of delivery, and birth orders. Characteristics of the children included in the study are summarized in Table 1.

**Table 1:** Characteristics of children aged 4–14 years diagnosed with Type 1 DM who were followed in the Endocrine and Metabolic Diseases Outpatient Clinic (cases) and of children who came to the General Pediatric Outpatient Clinic (controls) (N=248)

*Characteristics*	*Cases* *n (%)*	*Controls* *n (%)*	*p*
**Sex** (N=246)			
Female	63 (52.5)	54 (42.8)	0.130
Male	57 (7.5)	72 (57.1)	
Age at enrollment*, mean ± SD (N=246)	10.43 ± 3.31	7.48 ± 2.56	**<0.001**
Age at diagnosis**, mean ± SD (N=120)	6.30 ± 4.03	7.48 ± 2.56	**0.006**
**BMI range^+^** (percentile) (N=242)			
<5th	6 (5.1)	23 (18.9)	**0.006**
5th–84th	84 (71.2)	75 (61.5)	
85th–94th	18 (15.3)	11 (9.0)	
≥95th	10 (8.5)	13 (10.7)	
**Form of delivery** (N=244)			
Vaginal	40 (33.7)	43 (34.4)	0.897
Cesarean section	79 (66.3)	82 (65.6)	
**Birth weight^++^** (g) (N=242)			
<2500	15 (12.5)	12 (9.8)	0.515
2500–4000	97 (80.8)	105 (86.1)	
>4000	8 (6.7)	5 (4.1)	
**Interval between births** (years) (N=244)			
First child	49 (41.1)	54 (43.2)	**0.043**
<3	20 (16.8)	24 (19.2)	
3–6	26 (21.8)	37 (29.6)	
>6	24 (20.1)	10 (8.0)	
**Birth order** (N=244)			
First child	49 (41.2)	54 (43.2)	0.892
Second child	55 (46.2)	54 (43.2)	
Third or later	17 (12.6)	15 (13.6)	
**Duration of Type 1 DM** (years), mean ± SD (N=120)	4.16 ± 3.85		

* Age comparison with enrollment.

** Age comparison with Type 1 DM diagnosis age in cases, and enrollment age in healthy controls.

+ BMI range categorized for CDC chart. Less than 5th percentile: underweight; 5th–84th percentile: normal weight; 85th–94th percentile: overweight; ≥95th percentile: obese.

++ Birth weight classified for WHO. Low: <2500 g, Normal: 2500–4000 g, High: >4000 g. BMI: body mass index. CDC: Centers for Disease Control and Prevention.

Although no significant difference was found in the family history of DM in both groups, the family history of Type 1 DM was higher in the cases. Family characteristics of children are shown in Table 2.

**Table 2:** Family characteristics of study cases and controls (N=248)

*Characteristics*	*Cases* *n (%)*	*Controls* *n (%)*	*p*
**Residence** (N=243)			
Urban	34 (28.3)	54 (43.9)	**0.011**
District	83 (69.1)	62 (50.4)	
Rural	3 (2.5)	7 (5.7)	
**Family history of Type 1 DM** (N=240)			
No	107 (89.2)	119 (99.2)	**0.001**
Yes	13 (10.8)	1 (0.8)	
Maternal history of GDM* (N=188)			
No	72 (81.9)	86 (86.9)	0.264
Yes	17 (19.1)	13 (13.1)	
**Education level of mothers** (N=239)			
High school or higher	67 (56.8)	73 (60.3)	0.577
Lower than high school	51 (43.2)	48 (39.7)	
**Education level of fathers** (N=222)			
High school or higher	58 (53.7)	72 (63.2)	0.153
Lower than high school	50 (46.3)	42 (36.8)	

* This question was asked only to those who had an OGTT (oral glucose tolerance test) test during pregnancy, and nine mothers who had DM before pregnancy were also considered to have GDM (gestational diabetes).

Mothers who smoked during pregnancy constituted 13.3% of the cases and 14.4% among the controls. No statistically significant difference was found in the number of cigarettes mothers smoked per day and the duration of the smoking period during pregnancy and lactation between groups. Parents who were smokers were 54.7% among the cases and 59.5% in the controls (p=0.023). The mean number of cigarettes parents smoked at home per day before (3.28 ± 4.90) diagnosis was significantly higher in the cases than in the controls (2.12 ± 3.29). Table 3 summarizes the maternal smoking status during pregnancy and lactation, and the smoking at home of the parents.

**Table 3:** Maternal smoking during pregnancy and lactation, and parents’ daily household smoking, of study cases and controls (N=248)

*Tobacco smoking status*	*Cases*	*Controls*	
	*% (95% CI)*	*% (95% CI)*	*p*
**During pregnancy** (N=245)			
Smoking	13.3 (7.8–20.0)	14.4 (8.4–20.9)	0.925
No smoking	86.7 (80.0–92.2)	85.6 (79.1–91.6)	
Cigarettes per day, mean ± SD	4.81 ± 3.10	4.33 ± 4.39	0.281
Duration of smoking (months), mean ± SD	8.31 ± 1.66	7.17 ± 2.68	0.164
**During lactation** (N=238)			
Smoking	13.7 (7.3–20.6)	14.0 (8.3–20.4)	0.933
No smoking	86.3 (79.4–92.7)	86.0 (79.6–91.7)	
Cigarettes per day, mean ± SD	5.67 ± 3.18	5.69 ± 4.50	0.735
Duration of smoking (month), mean ± SD	13.73 ± 8.92	13.24 ± 9.93	0.955
**Household** (N=238)			
Smoking	54.7 (45.9–63.4)	59.5 (50.9–68.5)	**0.454**
No smoking	45.3 (36.6–54.1)	40.5 (31.5–49.1)	
Parents’ cigarettes per day smoked at home*, mean ± SD	3.28 ± 4.90	2.12 ± 3.29	**0.039**

* The number of cigarettes smoked daily at home by the parents in the control group while being included in the study was compared with the number of cigarettes smoked per day at home by the parents in the case group before their child was diagnosed with Type 1 DM.

When the mean of the HbA1c % levels at the diagnosis according to the smoking status of the parents of the children with Type 1 DM was examined, the mean HbA1c levels of the children whose parents were smokers at diagnosis was 10.94 ± 2.49, while the average of the non-smokers was 10.38 ± 2.77. However, this difference was not significant (p=0.298).

In logistic regression analysis adjusted for age and sex, we calculated the adjusted odds ratio (AOR) to determine if the likelihood of having Type 1 DM was associated with parental smoking at home, which was found to be 1.08 times higher for each additional cigarette smoked daily (p=0.039). No significant increase in odds was observed in the maternal smoking status during pregnancy and lactation (Table 4).

**Table 4:** Regression analysis comparing different variables related to smoking, for study cases and controls (reference) (N=248)

*Variables*	*n*	*AOR (95% CI)*	*p*	*R^2^*
Maternal smoking during pregnancy (no vs yes)	245	0.90 (0.43–1.89)	0.078	0.054
Duration of maternal smoking during pregnancy (months)	245	1.01 (0.92–1.11)	0.854	0.054
Daily number of cigarettes smoked by mother during pregnancy	245	1.00 (0.88–1.12)	0.948	0.054
Maternal smoking during lactation (no vs yes)	238	1.01 (0.48–2.14)	0.982	0.048
Duration of maternal smoking during lactation (months)	237	1.00 (0.96–1.05)	0.906	0.049
Daily number of cigarettes smoked by mother during lactation	236	0.99 (0.89–1.11)	0.853	0.049
Parental smoking at home and the same close environment (no vs yes)	238	0.82 (0.49–1.39)	0.464	0.058
Daily number of cigarettes smoked by the parents at home	225	1.08 (1.00–1.15)	**0.039**	0.088

AOR: adjusted odds ratio, adjusted for age and sex.

### Sensitivity analyses

Additional adjustment for early life variables did not modify the identified associations, and the results remained even when missing covariate data, as shown by a complete case analysis with and without covariate adjustment. Combined data on maternal smoking during pregnancy and lactation did not show higher odds of being exposed to maternal smoking in children with Type 1 DM. In all models, children with Type 1 DM had higher odds of being exposed to an increased number of cigarettes smoked by parents at home (Supplementary file Table 1). We also performed a *post hoc* analysis which showed that we achieved an effect size of 0.28 and a power of 0.59.

## DISCUSSION

There are studies underlining that passive smoking has an effect on diabetes and diabetes management, including HbA1c^18^ and blood glucose control^19^. This study examined the relationship between SHS and Type 1 DM, and has identified possible important modifiable risk factors for Type 1 DM, notably parents’ household smoking and the number of cigarettes daily smoked at home.

### Parental smoking at home

Studies investigating the relationship between passive smoking and risk of Type 1 DM, are few and the possible mechanisms have not been clarified^8,20-23^. Regarding the mechanism of passive smoking promoting diabetes metabolism, some studies suggest that nicotine affects the function of islet cells and insulin^24^. Moreover, nicotine promotes the degradation of insulin receptor substrate-1 (IRS-1) by activating AMP-activated protein kinase α2 in adipocytes to increase lipolysis in adipose tissue^25^. Through these mechanisms, passive smoking disrupts carbohydrate metabolism and promotes the development of abnormal glucose metabolism^19^.

We found increasing odds of exposure to a higher number of cigarettes smoked at home by parents among children with Type 1 DM. Therefore, the increase in risk might be dose-dependent. Further studies should focus on the relationship between Type 1 DM and the dose of SHS besides evaluating SHS smoking status only. A 10-year follow-up prospective study investigating the relationship between passive smoking and Type 2 DM showed that women who never smoked and women who were exposed to SHS from spouses had a higher risk of diabetes compared to women who were not exposed. In addition, passive smoking at home, estimated from the number of cigarettes smoked per day by the spouse in the study, was associated with a higher likelihood of developing diabetes^26^. Another study evaluating the association between smoking and metabolic parameters in patients with Type 1 DM showed that a significant proportion of patients were active or passive smokers, and suggested a relationship between smoking and adverse metabolic profile^22^.

The United States National Health and Nutrition Examination Survey (NHANES 1999–2010) demonstrated that SHS is correlated with obesity and worse glycemic parameters^27^. Our study did not compare SHS and glycemic parameters, except HbA1c. We observed that children with Type 1 DM whose parents smoked before diagnosis had slightly higher HbA1c levels at the time of diagnosis compared to non-smokers. However, this result was not statistically significant, but it may be evaluated more efficiently in studies with higher numbers of participants in which the level of cotinine could be measured.

### Maternal smoking during pregnancy and lactation periods

We found no association between Type 1 DM and neither maternal smoking during pregnancy or lactation periods. Nevertheless, several studies showed that maternal smoking during pregnancy has a triggering effect^28^, no effect^29^, and even a protective effect^30-32^ on the risk of Type 1 DM in the offspring. Surprisingly, prospective studies suggest a reduced risk of Type 1 DM in the offspring of mothers who smoked during pregnancy^7,33^. According to an animal study, smoking may help prevent the development of autoimmune diabetes by preserving pancreatic insulin content and encouraging anti-inflammatory mechanisms^34^. This mechanism is thought to be related via the ‘nicotinic anti-inflammatory pathway’^35^. However, the mechanisms are not fully elucidated, and human studies are inconclusive. On the other hand, these results should be interpreted cautiously as smoking status was self-reported, as in our study, and there was a large amount of missing data in certain studies^30^. Moreover, the selection of the control group in our study from a tertiary hospital may have caused a difference (and bias) in the results.

Similarly, a meta-analysis study including 23 studies showed that maternal smoking reduces the risk of Type 1 DM in the offspring. However, only one of the included studies^36^ measured the cord blood cotinine level, which indicated prenatal smoking exposure. In addition, the bias in the research due to the heterogeneity of the studies is underlined^37^.

### Limitations

There are some limitations in the study. First, passive smoking exposure in children was not measured with biological samples, and it was estimated from the self-report of parents. Therefore, it may have been underreported. Second, the reporting of SHS exposure is subject to recall bias. In addition, the selection of the control group from a tertiary hospital may have created a bias in the representation of this group. At the same time, it enabled a similar education distribution in both parents of the two groups.

Given that a significant portion of the control group visited the clinic due to upper respiratory tract infection, and considering the established correlation between passive smoking and upper respiratory tract infection, it is prudent to approach the study results with due caution. However, it is essential to acknowledge that selecting the control group exclusively from a tertiary hospital introduces potential bias in the study. Third, there may be response bias due to parents in both groups, particularly among the cases, who may have misrepresented their smoking status during pregnancy and lactation out of guilt. Fourth, we questioned maternal smoking during pregnancy and the lactation period, but we did not directly ask about paternal smoking in these two periods. Therefore, the results may not accurately reflect pregnancy or lactational exposure to smoking. Finally, those exposed to SHS at home may have many other risk factors for DM or factors that could lead to DM (reduced access to healthcare, lower level of education, socioeconomic status, etc.) that could affect the interpretation of the results.

Future studies with larger populations would increase the power of the study. They would enable the comparison of paternal smoking status besides the maternal smoking status, duration, the number of cigarettes smoked near the children per day, and in which place in the house they smoked (balcony or living room, etc.). Although we performed a sensitivity analysis, residual confounding factors may have been due to unforeseen variables and/or missing data.

The health-related effects of passive smoke are known to be linked with temperature, humidity, ventilation, depth of breathing, and distance from the smoker^6^. Another limitation is that these variables could not be evaluated in our study.

## CONCLUSIONS

Based on our results, children with Type 1 DM were more likely to be exposed to SHS. The fact that this risk factor is particularly modifiable can be an effective outcome for policy-makers in decision-making. SHS exposure should be emphasized as a milestone in the primary prevention of the disease, highlighting the need for intensified smoking prevention and cessation programs. Given the study’s limitations, it is crucial to recognize that prospective cohort studies involving larger populations will help to clarify the association between passive smoking and Type 1 DM.

## Supplementary Material



## Data Availability

The data supporting this research are available from the authors on reasonable request.
