# Supplementary material for: A retrospective evaluation of parental smoking and the risk of Type 1 diabetes in children
Source: Tob Induc Dis. 2024 Nov 21;22:10.18332/tid/195228. doi: 10.18332/tid/195228 (PMC11580006; doi:10.18332/tid/195228)
Supplement: Supplementary file 1 [file TID-22-180-s1.pdf]

Supplementary Table 1.

	Adjusted for sex and age				Adjusted for age				Adjusted for some confounders and specific variables (Model 1 adjusted for 9 variables)**				Adjusted for some confounders and specific variables (Model 2 adjusted for 4 variables)***			
**Factors**	***n***	**aOR (95% CI)**	***p***	**R^2**	***n***	**aOR (95% CI)**	***p***	**R^2**	***n***	**aOR (95% CI)**	***p***	**R^2**	***n***	**aOR (95% CI)**	***p***	**R^2**
Maternal smoking during pregnancy (no vs yes)	245	0.90 (0.43; 1.89)	0.078	0.054	245	0.93 (0.44; 1.94)	0.841	0.041	**171***	1.50 (0.26; 8.77)	0.650	0.218	239	1.07 (0.48; 2.40)	0.864	0.147
Duration of maternal smoking during pregnancy (month)	245	1.01 (0.92; 1.11)	0.854	0.054	245	1.01 (0.93; 1.11)	0.770	0.041	178	1.12 (0.98; 1.27)	0.091	0.203	239	1.04 (0.94; 1.15)	0.460	0.150
Number of cigarettes smoked per day by the mother during pregnancy	245	1.00 (0.88; 1.12)	0.948	0.054	245	1.001 (0.89; 1.13)	0.986	0.041	178	1.21 (0.97; 1.51)	0.085	0.205	239	1.03 (0.90; 1.17)	0.712	0.148

Maternal smoking during lactation (no vs yes)	238	1.01 (0.48; 2.14)	0.982	0.048	238	1.02 (0.48; 2.15)	0.963	0.038	**171***	1.72 (0.34; 8.69)	0.513	0.218	233	1.08 (0.49; 2.39)	0.852	0.142
Duration of maternal smoking during lactation (month)	237	1.00 (0.96; 1.05)	0.906	0.049	237	1.002 (0.96; 1.05)	0.919	0.038	173	1.03 (0.97; 1.09)	0.339	0.198	232	1.01 (0.96; 1.06)	0.748	0.144
Number of cigarettes smoked per day by the mother during lactation	236	0.99 (0.89; 1.11)	0.853	0.049	236	0.996 (0.89; 1.11)	0.939	0.038	172	1.12 (0.95; 1.32)	0.190	0.213	231	1.01 (0.90; 1.14)	0.839	0.148
Parental smoking at home and the same close environment (no vs yes)	238	0.82 (0.49; 1.39)	0.464	0.058	238	0.85 (0.51; 1.44)	0.547	0.043	**171***	0.84 (0.42; 1.70)	0.623	0.218	235	0.80 (0.46; 1.38)	0.420	0.157
Number of cigarettes smoked per day by the parents at home	225	1.08 (1.004; 1.15)	0.039	0.088	225	1.08 (1.01; 1.16)	0.032	0.076	167	1.15 (1.04; 1.27)	0.009	0.245	223	1.08 (1.002; 1.15)	0.045	0.206
									***Items written in bold were analyzed at the same time**							

									****Adjusted for age, sex, birth interval, residence, birth weight, history of GDM, history of family type 1 DM, mother's education level, delivery mode**	*****Adjusted for age, sex, birth interval, history of family type 1 DM**

© 2024 Cicekli I. and Durusoy R.
